# Optogenetic Methods to Investigate Brain Alterations in Preclinical Models

**DOI:** 10.3390/cells11111848

**Published:** 2022-06-05

**Authors:** Marco Brondi, Matteo Bruzzone, Claudia Lodovichi, Marco dal Maschio

**Affiliations:** 1 Institute of Neuroscience, National Research Council-CNR, Viale G. Colombo 3, 35121 Padova, Italy; marco.brondi@unipd.it (M.B.); claudia.lodovichi@unipd.it (C.L.); 2 Veneto Institute of Molecular Medicine, Via Orus 2, 35129 Padova, Italy; 3 Department of Biomedical Sciences, Università degli Studi di Padova, Via U. Bassi 58B, 35121 Padova, Italy; matteo.bruzzone@studenti.unipd.it; 4 Padova Neuroscience Center (PNC), Università degli Studi di Padova, Via Orus 2, 35129 Padova, Italy

**Keywords:** optogenetic methods, brain disorders, preclinical models

## Abstract

Investigating the neuronal dynamics supporting brain functions and understanding how the alterations in these mechanisms result in pathological conditions represents a fundamental challenge. Preclinical research on model organisms allows for a multiscale and multiparametric analysis in vivo of the neuronal mechanisms and holds the potential for better linking the symptoms of a neurological disorder to the underlying cellular and circuit alterations, eventually leading to the identification of therapeutic/rescue strategies. In recent years, brain research in model organisms has taken advantage, along with other techniques, of the development and continuous refinement of methods that use light and optical approaches to reconstruct the activity of brain circuits at the cellular and system levels, and to probe the impact of the different neuronal components in the observed dynamics. These tools, combining low-invasiveness of optical approaches with the power of genetic engineering, are currently revolutionizing the way, the scale and the perspective of investigating brain diseases. The aim of this review is to describe how brain functions can be investigated with optical approaches currently available and to illustrate how these techniques have been adopted to study pathological alterations of brain physiology.

## 1. Introduction

Understanding how the brain encodes even a simple sensory stimulus and generates a functional outcome is a challenging task because of the complexity of the underlying neuronal structure and function. Neuronal networks, along with other non-neural elements, form intricate local and global functional assemblies displaying coordinated and interdependent activity among different brain areas. These neuronal circuits show distinctive wiring diagrams, characteristic electrophysiological features, neurotransmitter specificity and distinct molecular mechanisms. Moreover, the structural and functional interactions supported by these components are tightly linked one to the other, and from this relationship emerge, in normal conditions, the fundamental brain capabilities of communication and information processing. Evidence from clinical and pre-clinical studies indicates that the normal level of coordination in the activity of neurons within the circuit is altered in several neurological and neuropsychiatric disorders, resulting in suboptimal or significantly impaired performances. It is noteworthy that in many cases these alterations can already appear in the prodromal phase of the disease. As a consequence, studying the brain functions, in normal and pathological conditions, requires multiscale approaches, from micrometers to several centimeters and from milliseconds to days. 

Moreover, it is crucial to consider different natural functional states, such as resting, sleep and sensory stimulation, as well as considering the animal model engaged in active tasks of different kinds. Traditionally, this has been achieved by means of electrophysiological recordings such as electroencephalogram (EEG) and Local Field Potentials (LFP), and/or recording from single or a limited number of cells. These methods are capable of providing a general picture of the ongoing dynamics but lack the possibility to capture extended network dynamics with the desired resolution, and to dissect the contribution of the different neuronal components is only partially possible. To integrate this information, these approaches have been complemented with different paradigms of neuromodulation relying on electrical stimulation or pharmacological treatments, aiming at isolating the role of different neuronal substrates or molecular mechanisms. However, this goal is challenging to achieve as pharmacological approaches can miss the target-specificity required as they typically present a broad spatio-temporal action spectrum, preventing investigation of a specific subpopulation of cells with precise resolution. In addition, they may exhibit undesired side effects, limiting the strength of the conclusions one can draw from such approaches. More recently, taking advantage of light-based activity reporters and actuators, optical methods have extended and refined the range of investigation and the number of units detectable along with their position. In the last 15 years, the amount and specificity of information retrieved from neuronal population recordings has substantially increased, leveraging improved cellular specificity. This result has been obtained with refined genetic strategies which allow targeting genetically-encoded activity reporters based on fluorescent proteins to identified neuronal or cellular populations. Along with the design of increasingly more refined genetically encoded molecular tools, suitable technologies and optical methods have been developed and are continuously optimized to extend the investigation in the depth, including subcortical brain areas, and in the width, encompassing larger neuronal populations over wide regions of the brain. To fully characterize the components of neuronal circuits and their role in network dynamics, genetically encoded activity reporters have been complemented with genetically-encoded modulators of cellular activity. Most of these light-activated molecules have been developed for non-invasively controlling the membrane potential of the target cells and, with this, their firing properties. Optogenetic stimulation relies indeed on the light-dependent gating of specific ion channels that once triggered and override ongoing cellular activity. Once the cells have been conferred with this kind of light-dependent membrane permeability, depending on the molecule (ion channel/pump), on its particular spectral sensitivity and on its intrinsic ion specificity, it is possible to modulate in a bidirectional manner, depolarizing or hyperpolarizing the cell membrane potential. The impact of the optogenetic intervention is sufficient to modulate the firing rate of a number of target cells at once, during repeated cycles of perturbations, or for prolonged time windows, according to the experimental protocol and to the biophysical properties of the selected opsin. In comparison with electrical stimulation, this approach is characterized by a largely improved cellular specificity and by an extremely refined spatial selectivity, as far as proper illumination methods and transgene expression approaches are adopted. A growing number of researchers currently use optical methods to gain insights into the circuit dynamics underlying distinct brain mechanisms and functions, either by recording the ongoing circuit activity in different scenarios and/or by assessing the impact of optogenetic manipulations of specific neuronal populations on circuits activity and, more generally, on brain function. On the other side, considering the study of neurological or genetic brain diseases, these approaches are still in their take-off phase, despite their recognized potential. The aim of this review is then to present the most common optical technologies available for the analysis of neurodegenerative, psychiatric and neurological disease models and to showcase a series of applications in the study of different pathologies. 

## 2. Molecular and Hardware Tools to Optically Record Brain Activity

### 2.1. Molecular Tools to Optically Record Brain Activity

#### 2.1.1. Genetically Encoded Calcium-Based Indicators (GECI)

**Figure 1:**
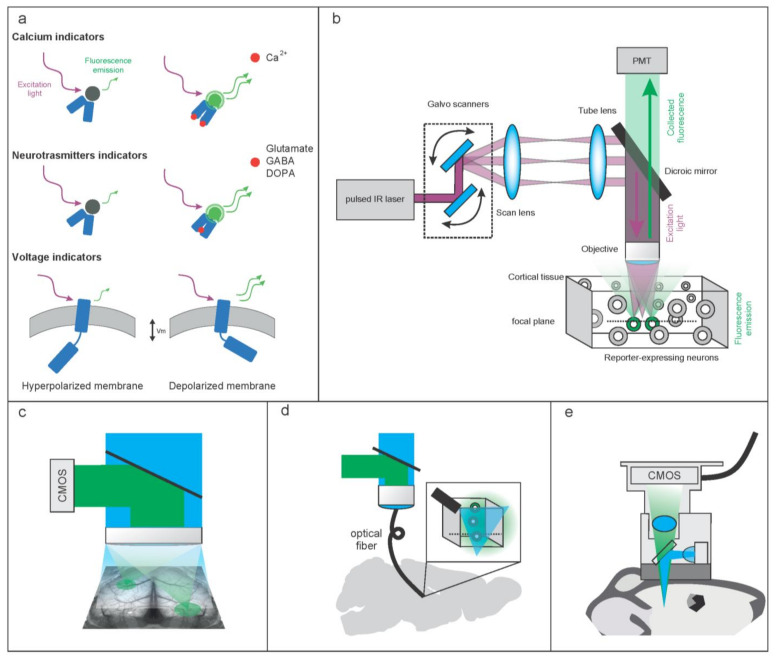
Fluorescence-based methods for in vivo imaging of neuronal physiology. (**a**) Genetically encoded fluorescent reporters of calcium (top), neurotransmitters (middle) and membrane potential (bottom) are designed to display variations in the fluorescence yield (green) as a consequence of the binding of the analyte to the reporter or changes in the electrical potential across cell’s membrane. (**b**) Generic scheme of scanning fluorescence microscope: excitation light (purple, infrared in the case of two-photon laser source) enters the sample through an optical access and is confined in a diffraction limited spot in the tissue expressing the genetically-encoded reporter (circles representing neuronal cells). The excitation beam is scanned across the tissue using a pair of galvanometric mirrors (or equivalent devices such as resonant scanners or acousto-optical deflectors) pointing sequentially the diffraction limited spot to fixed coordinates in a raster. Fluorescence from cells expressing the reporter (green) is collected as it irradiates outside the tissue, as the excitation beam stops at each position in the scanning sequence. An image is then reconstructed using a bucket detector (e.g., photomultiplier tube). This scheme applies to two-photon imaging of deep structures. (**c**) In the case of wide-field imaging, the excitation light homogeneously illuminates the tissue and the fluorescence is collected with a spatially-resolved sensor. (**d**) In fiber photometry approaches, fluorescence is excited in the proximity of a penetrating optical probe (fiber) inserted in the tissue. The excitation light exits the tip of the probe and the local fluorescence intensity is recorded through the same optical fiber without forming an image. (**e**) Miniscopes are miniaturized fluorescence microscopes designed to be implanted on the animal’s skull, allowing for recordings during unrestrained behavior. Excitation light is delivered inside the tissue through an optical fiber or an optical access to the dorsal brain surface. The fluorescence is collected by a spatially resolved sensor in order to form an image. All these methods are capable of recording data sequentially, allowing for a dynamic (i.e., extended in time) characterization of neuronal physiology.

Neuronal activity causes rapid changes in intracellular free Ca^2+^ levels [1]. Based on this principle, genetically encoded fluorescent Ca^2+^ indicators (GECI) have been designed to optically report the increase in the calcium level associated with the firing activity of neurons (Figure 1a). This approach enables the recordings of large populations of cells expressing the indicators. Furthermore, by genetic engineering modifications, the sensors can be targeted to selective cell types or sub-cellular compartments to perform Ca^2+^ imaging in specific domains [2,3,4].

The most widely used genetically encoded Ca^2+^ sensors, GCaMPs, are based on circularly permutated green fluorescent protein (cpGFP). The N-terminus of cpGFP is linked to the M13 fragment of the light chain of myosin kinase, which is a target sequence for Calmodulin (CaM), hence the name calmodulin binding peptide (CBP), whereas the C-terminus of cpGFP is linked to CaM [5]. Ca^2+^-dependent conformational changes of CaM, also through its interactions with CBP, propagates to the rest of the protein modulating the GFP chromophore environment, resulting in increased fluorescence quantum yield. Through iterative rounds of protein modifications and optimization, several versions of GECI, incorporating different fluorophores [6,7] with increased signal-to-noise ratio (SNR), emission spectra and distinct kinetics and dynamic range, have been generated, allowing for accurate recording of neuronal activity with high spatial and temporal resolution [6,8,9]. Cytosolic GECI also allows the measurement of Ca^2+^ fluctuations in fine neuronal processes, such as dendrites and spines, when the labelling is sparse. In conditions of dense labelling, the neuropil also represents an obstacle to clearly measure Ca^2+^ activity in individual soma, which are often enwrapped by neuronal processes. In addition, dendritic arborization in superficial brain layers can act as a shield, preventing the monitoring of the Ca^2+^ dependent fluorescence changes in deeper layers, where cell bodies are located. To overcome this limitation, GECI have been efficiently targeted to the cell body or the nucleus in zebrafish and fly [10,11,12]. In mammals, targeting GCaMP to the cell body was more challenging. However, recently, a new engineered version of GcaMP tethered to ribosomes was reported to faithfully track Ca^2+^ dynamics with reduced contamination from the neuropil. With this sensor, the fluorescence signal derives mostly from the soma, which is enriched in ribosome with respect to the neuropil [3]. In addition, by screening several artificial and natural peptides, two motifs were identified, that when fused to GCaMP allow it to be expressed within 50 μm from the nucleus. These versions of GCaMP track Ca^2+^ dynamics with reduced background noise due to the reduction of the signal contamination from the neuropil. This results in higher SNR, both in mice and fish, and in the possibility, under favorable circumstances, to track suprathreshold dynamics with single action potential (AP) resolution [2] (see Table 1).

In fact, although calcium imaging proved to be an excellent technique to probe neuronal dynamics from cellular to network level, calcium events in neurons are a good proxy of their spiking activity only in certain working conditions. Indeed, molecular kinetics of GECI are intrinsically too slow to allow for accurate and temporally resolved tracking of network activity down to individual action potentials with single cell resolution over wide fields of view [13]. As a consequence, isolated spikes and/or individual action potential within bursting activity are poorly detected [6,14]. Moreover, along with the spiking activity, subthreshold oscillations of the membrane potential are important features governing the neuronal dynamics in the organization of behavior and in cognition [15,16]. Unfortunately, GECI affinity to calcium is mostly tailored to report relatively large changes in Ca^2+^ concentration associated with cell firing [6,9,17]. This renders these molecules practically unsuitable for detecting smaller calcium fluctuations that are totally “blind” to calcium-independent fluctuations of the membrane potential.

#### 2.1.2. Genetically Encoded Voltage Indicators (GEVI)

Genetically Encoded Voltage Indicators (GEVIs) have the potential to overcome the limitations of GECIs allowing for cellular resolution voltage imaging and recording the dynamics of a small population of neurons in a living organism [18]. The general molecular structure of a GEVI consists of a voltage sensing sub-domain (VSD) fused to a fluorescent protein or to a so-called fluorescence resonance energy transfer (FRET) protein couple (Figure 1a). The latter is a structure with two fluorescent proteins, an acceptor and a donor, whose respective absorption (acceptor) and emission (donor) spectra overlap. This allows for a non-radiative energy transfer between the two molecules resulting in a variation of their fluorescence emission. Energy transfer via FRET occurs indeed only when the two fluorophores are in close proximity, generally <10 nm, and the ratio between donor and acceptor fluorescence is referred to as the FRET signal [19].

A change in the membrane potential leads to a conformational re-arrangement of the GEVI structure, resulting in the increase or decrease of the FRET signal [18]. The appropriate choice of a suitable GEVI is thus subordinated to the associated spectral and biophysical properties [18]. In all cases, compared to the most soluble GECIs, GEVIs are expressed only in the cell membrane to be sensitive to the variation in membrane potential, limiting the number of molecules contributing to the generation of the detected signal. Moreover, the changes in fluorescence due to the variation of the membrane potential are rather small, by a few percent of the baseline compared to fold increases observed with GECI. In addition, as a consequence of the spatial confinement of GEVI on the cell surface, motion artefacts may be even more detrimental to the quality of imaging compared with GECI and signal contamination from background structures (e.g., neuropil) and could represent an important issue [20]. A sparse labelling strategy, limiting the number of cells expressing the voltage reporter, could therefore be an effective method to minimize signal contamination from other cells and to image membrane voltage changes along the dendritic tree.

From the hardware point of view, classical raster scanning imaging systems are sub-optimal for GEVIs mainly as a consequence of slow scanning speed compared to the fast GEVI dynamics which reflect the rapid fluctuations of the membrane potentials. Given the relatively low SNR associated with GEVI imaging, high excitation power is typically required when compared with GECI [18]. Phototoxicity and indicator bleaching processes are thus to be seriously considered when using GEVI. Acousto-optic deflector-scanners (AODs), in combination with the engineering of the excitation wave-front, are efficient technical approaches suitable to achieve fast neuronal population voltage imaging and increase excitation efficacy in order to extract spiking and subthreshold signal in individual neurons with millisecond temporal resolution [21].

From the perspective of network neuroscience, GEVI are interesting and fast developing tools to be applied at different scales: from the cortex-wide meso and macroscopic data collection (one and two-photon) in behaving animals, to more local, microcircuit-oriented applications. Furthermore, extending GEVI applications to finer subcellular compartments allow for spine voltage imaging. GEVI-directed expression to cell classes or cortical layers of interest leverages recent intersectional genetic approaches, extends the application of these tools beyond classic electrophysiological methods and opens the field of so-called “all optical electrophysiology” [22].

In conclusion, GEVI are an exceptional tool for network neuroscience, still amenable to large improvements possibly beyond GECI capabilities and thereby allowing, in perspective, more precise dissection of underlying mechanisms such as neurodegenerative diseases, developmental dysfunctions or basic circuit dynamics associated with perception and behavior.

#### 2.1.3. Imaging Neurotransmitters and Neuromodulators

Cognitive functions at all levels relies on fine tuning of neurotransmitters and neuromodulators (NT/NM) release, both in time and space [23,24]. Even subtle alterations of this precisely orchestrated chemical environment may result in overt dysfunctions observable at the behavioral level [25,26,27,28,29,30]. While activity of even large neuronal populations can be monitored in vivo with high precision using GECI or GEVI, tracking the dynamics of NT/NM has become possible with comparable resolution and accuracy only recently thanks to the development of specific NM/NT biosensors [31].

The general design for NT/NM reporters, similar to the one described for GECI and GEVI, comprises a fluorescent component that can be a single fluorescent protein or a FRET couple (Figure 1a). The fluorescent moiety is linked to a “sensing domain” (SD), capable of binding the NT/NM. As in the case of GECI and GEVI reporters, the fluorescence emission is modulated by a conformational change of the sensor triggered by the interaction between the SD of the sensor and its ligand, that in turn leads to an increase/decrease of fluorescence protein (FP) quantum yield or affects the FRET coupling, hence its signal [31]. Presently, two alternative molecular designs are available for the SD; one incorporates a periplasmic binding proteins (PBPs) cloned from different prokaryotic strains [32], and the other leverages G-protein coupled receptors (GPCRs) from either prokaryotes or eukaryotes [33]. PBPs are proteins secreted in the periplasmic space, such as Venus Flytrap Domain (VFTD), that recognize specific ligands [34]. The circularly permutated fluorescent protein (cpFP) is typically inserted in a flexible region of VFTD in order to propagate the conformational change to the chromophore core and thus modulating cpFP quantum yield [35]. Alternatively, each protein of a FRET pair is inserted on the opposite sides of the VFTD, and when the ligand is bound, the two fluorescent proteins are brought in close proximity enabling the FRET effect [36]. NT/NM biosensors based on GPCRs are formed by GPCRs linked to cpFP or a FRET couple in a similar design compared with PBP-based sensors. These GPCR-activation-based (GRAB) sensors convert the conformational change induced by the ligand in the GPCR into a fluorescence change in the cpGFP [31,37].

Several genetically encoded sensors for neurotransmitters and neuromodulators are currently available. To monitor the kinetic mechanism of glutamate binding, a sensor based on the bacterial periplasmatic glutamate/aspartate binding protein (GluBP) fused to the circularly permutated enhanced green fluorescence protein (cpEGFP), SFGluSnFR, was developed [38]. Variants of SFGluSnFR indicated as iGlu_m_, iGlu_h_, iGlu_l_ [39] and the fast (iGlu_f_) and ultrafast (iGlu_u_ [40] have a broad range of sensitivity and high temporal and spatial readouts suitable for in vivo applications. In addition, multicolor variants of Glutamate biosensors have been obtained, replacing the GFP FP with blue- (SF-GluSnFRA184V [41]) or red-shifted variants (R-iGluSnFR1 and R-ncp-iGluSnFR1 [42]). In contrast, with the more common NT/MT biosensors, in which fluorescence increases upon ligand binding, R-iGluSnFR1 reports glutamate binding with a reduction of FP quantum yield [39,42].

The genetically encoded sensor for GABA, iGABASnFR [43], is based on cpsfGFP and the Pf622 VFTD PBP protein. This protein is targeted to the cell membrane via an N-terminal immunoglobulin secretion signal and a C-terminal transmembrane domain of the platelet-derived growth factor receptor (PDGFR).

Acetylcholine dynamic can be tracked with M1-cam5, a FRET-based Acetylcholine biosensor based on excitatory M1 muscarinc receptors [44], whereas GACh2.0 [45] is a single FP-based reporter, based on a chimeric muscarinic and adrenergic β2 receptor, which has the sensitivity, kinetics and photostability suitable for investigating Acetylcholine signals in vitro and in vivo. 

As for dopamine, GRAB_DA_ [46,47] is a dopamine biosensor based on the D1 receptor and cpEGFP, showing fast fluorescence increase kinetics and no apparent interaction with the endogenous GCPR signaling pathway. dLight1 and dLight1.2 [48,49] are two dopamine biosensors incorporating the human D1 receptor, recently expanded into a palette of reporters, with different affinity and spectral properties. dLights do not interfere with endogenous GPCR signaling or cAMP pathways. Both GRAB_DA_and dLights show no internalization, allowing for stable expression suitable for chronic experiments.

Sensors for norepinephrine (NE) are GRAB sensors, in which cpEGFP is inserted in the third loop of the adrenergic receptor alfa 2 (α2AR). This family of sensors for NE, indicated as GRAB_NE_, exhibit high sensitivity, specificity and relatively fast kinetics, being suitable for revealing changes in NE in different behavioral conditions in zebrafish and mice [50]. GRAB_NEs_ are not exclusively selective for norepinephrine, showing significant affinity with epinephrine [50].

Genetically encoded sensors are also available for opioid, ATP and glycine. The sensor for opioids, called OR sensor [51], is based on a nanobody which recognizes only activated opioid receptors and is fused to EGFP. This opioid biosensor enables differentiation of the activation of opioid receptors by endogenous compounds with respect to exogenous molecules.

A ratiometric biosensor for ATP, named ATeam3.10, is a FRET-based sensor, developed from the bacterial ε subunit of FoF1-ATP synthase flanked by a FRET pair. In order to detect extracellular ATP, the sensor is endowed with an immunoglobulin K leader sequence and a transmembrane anchor domain from the PDGFR, resulting in the decoration of extracellular aspect of the plasma membrane [52]. The sensor iATPSnFR is similarly designed but, instead of a FRET pair, a single sfGFP is inserted between two alfa helices of the ε subunit of FoF1-ATP synthase. To optimize the expression of the sensor on the cell surface, EGFP was replaced with SFGFP and additionally engineered to reduce sensor dimerization. The resulting iATPSnFR sensor presents an efficient cell surface trafficking and a ΔF/F of about 25% [53]. Recently, Wu et al. [54] developed a new ATP sensor, GRAB_ATP1.0_. This sensor is characterized by high temporal resolution (subseconds) and high affinity (tens of nanomolar) for ATP. These features make possible to track ATP dynamics at cellular and subcellular level in vitro and in vivo, in different models, i.e., zebrafish and mouse, providing a versatile tool to follow ATP kinetics in basal conditions and in response to stimuli, both in physiological and pathological conditions. 

Recently a FRET-based sensor for glycine, named GlySF, was developed using the Atu2442 protein from *Agrobacterium tumefaciens.* Atu2422 binds to glycine, serine and GABA. However, engineering the Atu2422 binding site resulted in a Glycine specific mutant named AYW, which was inserted between EGFP and mVenus FPs [55]. Unfortunately, in vivo application of GlyFS is still missing due to the lack of an appropriate extracellular targeting strategy.

The sensor for Serotonin is also a GRAB sensor, where cpGFP is inserted in the third loop of the serotonin receptor, 5HT2C. Mutagenesis and screening of different versions of the 5HT2C-based sensor, in particular of the linker regions and cpGFP, resulted in a sensor named GRAB_5HT1.0_ [56], characterized by high affinity, specificity and relative fast kinetics in tracking endogenous physiological release of serotonin. These features, as demonstrated, made this sensor suitable in detecting the endogenous release of serotonin in various animal models, i.e., Drosophila and mice, during distinct behavioral states and tasks (see Table 1) [56].

**Table 1:** Main properties of genetically encoded fluorescent reporters of endogenous biomolecules. Affinity (Kd), dissociation time constant (Koff), relative peak change in fluorescence over baseline signal (ΔF/F peak %), construct availability and relative reference (ref.) are reported for Calcium (green), Dopamine (red), Norepinephrine (orange), GABA (cyan), Serotonin (yellow), Glutamate (grey), Acetylcholine (purple), ATP (pink) and Adenosine (violet).

	Reporter	Analyte	Kd (nM)	Koff (ms)	ΔF/F0 Peak %	Available @	Ref.
	GCaMP6s	Calcium	147	1796	1680	Addgene	[9]
	GCaMP6f	Calcium	375	400	1314	Addgene	[9]
	jGCaMP7s	Calcium	68	1260		Addgene	[6]
	jGCaMP7f	Calcium	150	270	3100	Addgene	[6]
	jGCaMP8f	Calcium	334	27	7880	Addgene	Janelia
	jGCaMP8m	Calcium	108	55	4570	Addgene	Janelia
	jGCaMP8s	Calcium	46	272	4950	Addgene	Janelia
	dLight1.1	dopamine	330		230	Addgene	[48]
	dLight1.2	dopamine	770	90	340	Addgene	[48]
	dLight1.3b	dopamine	1680		930	Addgene	[48]
	GRAB_DA_1m	dopamine	130	700	90	Addgene	[46]
	GRAB_DA_2m	dopamine	90		340	Yu Long Li lab	[47]
	GRAB_DA_1h	dopamine	10	2500	90	Addgene	[46]
	GRAB_DA_2h	dopamine	7		280	Yu Long Li lab	[47]
	GRAB_NE_1h	norepinephrine	83	2000	130	Yu Long Li lab	[50]
	GRAB_NE_1m	norepinephrine	930	750	250	Yu Long Li lab	[50]
	iGABASnFR	GABA	9000			Addgene	[43]
	GRAB_5HT1.0_	serotonin	22	3100	280	Yu Long Li lab	[56]
	iSeroSnFr	serotonin	1500		250	Tian lab	[57]
	iGluSnFR	glutamate	4900	92	100	Addgene	[41]
	iGlu _f_	glutamate	137,000	2.1		Addgene	[40]
	iGlu _u_	glutamate	600,000	700		Addgene	[40]
	iACHSnFR	acetylcholine	1300		1200	Addgene	[58]
	GACh2.0	acetylcholine	2000	3700		Yu Long Li lab	[45]
	GRAB_ATP1.0_	ATP	45	9	1000	Yu Long Li lab	[54]
	iATPSnFR1	ATP	50		190	Addgene	[53]

#### 2.1.4. Directing Reporters Expression to Neuronal Populations of Interests

In order to optically record neuronal activity from specific neuronal populations, it is crucial to select the appropriate strategy for transgene delivery. One such approach involves the microinjection of a plasmid construct, carrying the coding and regulatory sequences for the indicator in the embryo of a recipient model organism (e.g., mouse). With the in-utero electroporation (IUE) method, the ventricle (s) of mouse embryos is injected with small amounts of solution containing the plasmid, whose migration to the neurogenic epithelium is driven by the application of a carefully designed electric field. This approach allows for the migration and incorporation of the exogenous DNA in neuronal progenitor cells while operating in laparoscopy so that gestation can follow normal progress after the intervention [59,60]. By selecting the appropriate embryonic developmental stage and using specific configurations of the electric field applied, it is possible to target progenitor cells that will migrate and become mature neurons into specific brain structures (e.g., hippocampus, visual cortex, motor cortex, cerebellum or individual cortical layers) in the adult mouse. This technique allows for the directed expression of the transgene even when the target region cannot be easily identified by genetic means, e.g., a specific promoter is missing. Approaches for transgene delivery based on recombinant adeno-associated viral vectors (rAAV) recently became more popular [61]. rAAV viral vectors incapsulating the indicator construct can be easily injected in the brain parenchyma of adult and newborn mice, resulting in efficient transduction upon infection. The transduction efficiency can indeed be tuned, selecting among several available rAAV serotypes and displaying different cellular host spectra with some serotypes showing a markedly restricted tropism toward neurons or astrocytes, for example. To further restrict the expression and target cellular sub-populations and/or regions, appropriate promoters can be selected and/or the viral injection procedure can be carefully adjusted to minimize diffusion. Combining classical genetic tools with rAAV allows for additional refinements of the targeting approach. Several transgenic mouse lines are indeed available, carrying, for example, GECI coding sequence knocked-in in genomic loci selected for high efficiency of transcription [62,63] and endowed with suitable promoters for cellular population selectivity. Although constitutive expression of the reporter is sometimes desirable, conditional and inducible expression strategies are often preferred, considering that GECI expression might start during early developmental stages, possibly interfering with the physiological process. In addition, long-term GECI expression might result in detrimental cellular functions or might lead to protein accumulation and deterioration of the imaging signal [64]. In this respect, knock-in GECI mice with “floxed” (flx) or “flipped” (flp) stop codons can be efficiently used as alternative to the constitutively expressing mouse lines. The flx/flp-ed codon can be conditionally removed, providing in “trans”-configuration the associated recombinase, typically via rAAV. Using this approach, the genomic GECI sequence can be regulated by generic and efficient promoters in “cis” design, with no selectivity for specific cell populations, whereas the construct in the rAAV vector is designed with the recombinase regulated by a cell population-specific promoter. In this way, even a small amount of recombinase produced by the infected cells and selected by the construct promoter is enough to trigger a sustained expression of the reporter only in those cells. This strategy is particularly useful when targeting relatively small structures that can be reached with the rAVV microinjection, avoiding undesired large reporter expression obtained, for example, by crossing the GECI mouse line with a mouse line expressing the recombinase in a specific cell population. An effective approach to control the timing of the transgene expression is the combination of rAAV and mouse lines with conditional transgenes. Furthermore, the activation of a transgene can be finely regulated by inducible rAAV. Alternatively, crossing a transgenic mouse line expressing a conditional trans-activator module (possibly floxed/flipped) with another mouse line, with the reporter of interest under the control of the trans-activator response cassette, allows for fine tuning of the expression timing and offers the possibility to transiently stop the transgene expression relying on the classic Tet-ON/Tet-OFF strategy [65]. However, this approach does not allow the restriction of the transgene expression to relatively small areas.

Some rAAV serotypes display marked retrograde/anterograde transport from the injection site, allowing for the labelling of structures afferent/efferent to a selected target region, a useful property in functional connectome studies [66,67]. Several providers are available for direct purchase of the most published constructs, and customization of the construct is generally easily accessible; unfortunately, the rAAV injection procedure is invasive, and may result in inhomogeneous expression levels or might require several injection sites to cover broad areas. Recently developed rAAV (AAV-PHPeB) show high efficiency in crossing the blood–brain barrier when injected in the blood stream, reducing the invasiveness of classical rAAV injections and extending the accessible volume of transduction virtually to the whole brain, but retaining the possibility to target genetically identified neuronal populations [68]. Additionally, combining different cargoes with different rAAVs allows for the simultaneous transduction of different cell types with specific reporters (e.g., GECI with separate spectral properties) within a given target area [69,70]. Alternative or complementary to transduction methods, several transgenic mouse lines are available nowadays, expressing different fluorescent reporters in specific brain structures or suitable for intersectional targeting approaches [63]. Although often extremely useful, some of these reporter mouse lines might show suboptimal expression levels, expression leakage from undesired cell types or even reporter-associated neuronal toxicity [64]. Considering the relative ease in crossing different lines compared with mouse models, zebrafish offer great flexibility in combining neurodevelopmental disorder models and calcium imaging approaches. Indeed, a large palette of zebrafish models of neurodevelopmental disorders are available [71] and can be efficiently combined with several reporter-expressing lines with specific features [72].

### 2.2. Hardware Tools to Optically Record Brain Activity

Fluorescence microscopy is a powerful and largely used approach in biology to analyze morphological and functional properties of cells in living tissues, as long as a sufficient amount of light can reach the target region within the tissue. Biological samples indeed typically absorb and scatter incident photons in the visible wavelengths. As a result, the thicker the biological sample, the higher the loss of incident light intensity at the focal point, resulting in a progressive degradation of contrast caused by the light scattering itself. In confocal microscopy, in which the incident light is focused to a diffraction-limited spot, and the emitted fluorescence photons are collected through a conjugated pinhole, the contribution of the scattered light coming from planes out of focus is partially rejected. However, this approach is efficient if the sample is not very thick (below 80–100 μm). Increasing the power of the incident light does not help to get a better image deeper in the tissue as this leads to deterioration of the signal retrieved as consequence of the increased background levels. Furthermore, this comes with significant disadvantages due to the photodamage of the fluorophores (photobleaching) and to the tissue caused by the absorption of visible light. One photon excitation (1PE) is therefore sub-optimal for deep tissue imaging, especially in living tissues. These major drawbacks of confocal microscopy have been overcome by multi-photon microscopy (MPM) which allows optical sectioning and improved depth penetration, favoring neuronal recording deep in the whole living brain. MPM is based on the principle that multiple photons of lesser energy can produce the same excitation that is caused by the absorption of a single photon of higher energy, when the multiphoton absorption occurs “at the same time” (∼10^−15^ s). This process named multi-photon (MPE) or two-photon excitation (2PE) was theoretically predicted by Maria Göppert-Mayer in 1931.

As a consequence of the inverse relation between photon energy and its wavelength, each of the incident photons in MPE will have longer wavelengths compared with that of a single photon capable of triggering the same absorption process on a given fluorophore. Since the absorption of each of the incident photon is probabilistic and independent, the higher the intensity of the incident light, the higher the probability of the absorption process. In 1PE, this relation is linear, whereas in MPE it is non-linear, scaling up with the power of the number of photons that can be absorbed simultaneously. Typically, in multi-photon excitation, incident light is shifted to the infrared part of the spectrum corresponding roughly to twice the wavelength of an equivalent 1PE process. Longer wavelength light has a dual advantage in imaging living tissues: it has less scattering and penetrates deeper in the tissue. As a result, the light travels across the biological sample, losing less power at the level of the focal spot and the fluorescence signal can be kept relatively constant through the volume, with a relatively smaller power increase. This represents a great advantage, also considering that that red-shifted or infrared light is considered less phototoxic. Furthermore, in MPE, fluorescence is maximal at the focal point and decreases with the distance according to a power relation (depending on the number of photons absorbed in the multi photon process) away from it along the light propagation direction. This property gives MPE an “intrinsic” optical sectioning; the vast majority of the fluorescence signal comes from a small region (typically in the order of femtoliters in two-photon excitation), whereas very little absorption of incident photons happens along the light path above and below the focal point [73]. To maximize the probability of absorption, lasers with pulsed emission are implemented for multiphoton excitation. Brief pulses of light (140–200 fs) at very high energy (nJ) are delivered to the tissue at a high frequency (80 MHz for most pulsed lasers) while keeping the average power delivered to the sample at acceptable levels. Switching from 1PE to MPE, in general, does not require the selection of alternative fluorophores and scanning techniques and equipment can be adapted. All the advantages of MPE in recording living samples prompted a rapid diffusion of MPM and triggered several technological advancements and alternative imaging techniques based on MPE. This is currently considered the technique most suitable for imaging the activity of large populations of neurons with cellular or subcellular resolution in living animals [74,75]. 

#### 2.2.1. Hardware Configurations to Optically Record Brain Activity at High Resolution

The general configuration for optically reconstructing the neuronal activity in vivo is based on a layout for multiphoton microscopy and stems from a pulsed oscillator, ensuring high repetition rate and high energy emission of light pulses (Figure 1b). Concentrating the photons temporally in short high energy pulses and spatially in a diffraction limited spot is the key for enabling multiphoton absorption. The laser intensity is typically modulated with an internal or external component that can be a Pockels cell or an acoustic-optical modulator. The light beam path includes a series of optical components designed to obtain the required beam diameter and to direct the beam toward a scan-head based on a pair of small galvanic mirrors tilting along two orthogonal axes. The plane of the scanner is conjugated to the back aperture of the objective by a pair of lenses, called scan and tube lenses, respectively. In this way, the steering of the beam at the level of the scanner results in the movement of the excitation beam, typically with a raster scheme, on a 2D plane of the sample optically conjugated. Depending on the working principles of the mirrors, galvo–galvo or galvo-resonant, different frame rates can be achieved. With the galvo–galvo configurations, one can arbitrarily adjust the pixel dwell time, typically set between 1 and 4 us, ensuring the optimization of the number of emitted photons per pixel and the maximization of the signal-to-noise ratio. On the other hand, in the galvo-resonant scanning approach, the line scanning rate is fixed either to 8 kHz or 12 kHz, resulting in the maximization of the scanning speed and with typical pixel dwell times of 150–80 ns. Upon excitation, the fluorescence emitted is collected through the same objective and directed toward a set of extremely sensitive detectors, called photomultipliers tubes (PMTs), each converting photons of a specific spectral band in a proportional electrical signal. These detectors acquire the emitted fluorescence integrating the emitted light, both the ballistic and scattered components, generated at the excitation spot [76]. 

Optical recording of neuronal activity can be achieved with different approaches. In the imaging approach based on raster scanning schemes, the excitation beam visits all pixels along the scanning trajectory, agnostic of the position of cells or region of interest. Pixels belonging to cellular structures can be indeed a minority of imaged pixels, the remaining part being represented by background/neuropil structures. As a consequence, a relevant fraction of scanning time is spent acquiring signals of no or little interest, limiting the spatial and temporal resolution achievable. To overcome this limitation, a random-access sampling scheme is commonly adopted, where the excitation beam travels along a predefined trajectory, visiting the cells or the regions of interest and discarding the uninformative parts of the field of view. This scheme can be implemented by properly driving a galvo–galvo scanner but the mechanical inertia of these elements limits the maximal switching rate. For this approach, in fact, a different type of scanner, not based on moving mirrors but relying on acousto-optic deflectors (AODs) to steer the beam at different locations is more commonly used. AODs are crystal elements where the geometric distribution of the refractive index can be controlled with an appropriate sound wave, resulting in the modulation of light propagation properties. In this case, the switching time in the position of the excitation beam, typically in the order of a few us, is only limited by the propagation speed of the sound through the crystal and the size of the active region of the material. An AOD scanner is designed with two orthogonally oriented crystals, each coupled with a mechanical traducer driven by an electrical signal controlling the beam deflection along one axis of the plane. 

As an alternative to a layout with a single beam sequentially scanning the sample, in order to increase the acquisition bandwidth, parallel solutions based on spatial or temporal beam multiplexing have been developed. These approaches rely on the generation at the sample of a small set of simultaneous foci, each illuminating one specific point of the plane. This is achieved by splitting the original beam, either by a dedicated optical path or by using a structured illumination system to engineer the light distribution at the sample. In this design, the detection of the emitted signal originating at the different foci typically implements a pixelated detector as a camera or source separation algorithms to disentangle the different components [73,77]. 

#### 2.2.2. Methods for Extending the Imaging in the Third Dimension

Brain circuits in general do not lay on a single plane but rather span over a certain thickness of the tissue, and this has prompted the development of a series of methods to extending the imaging capability along the third dimension, allowing to sample from a volume rather than a single plane. The easiest implementation uses a mechanical piezoelectric actuator to move the objectives along the light propagation axis and in this way moves the actual depth in the tissue where the excitation beam is focused [78]. Such systems, synchronized with the continuous scanning along the X and Y directions, currently allow for capturing a volume as thick as 400 μm with a rate of 1–2 volumes per second [79]. Instead of mechanically moving a relatively heavy objective, that is always associated with undesired vibrations transferred to the sample, a number of so-called remote focusing methods [80,81] have been used, either to increase the commutation speed along the z-axis or to render the z-scanning scheme more flexible. In this alternative scenario, the objective is kept still and other optical components, placed upstream, are operated to change the geometry of the light wave front entering the objective, resulting in a change of the focal position. A few solutions envisage the reconfiguration of optical elements, such as lightweight mirrors or tunable lenses, by means of a proper electrical signal controlling their relative position or their geometrical properties. In the first case, a dedicated optical path for the excitation has to be integrated in order to optically conjugate the sample plane with the plane of a moving mirror, so that the shifting of the mirror causes an offset in the imaging position in the plane. On the other side, the layouts based on Electrically Tunable Lenses (ETLs) are generally more straightforward to implement as these elements are usually placed either on the back of the objective or in a plane optically conjugated to the objective back aperture at the level of the scanner or upstream to it [82,83]. These approaches can enable relatively fast refocusing of the imaging plane with typical settling times shorter than 5 ms when the system is driven with a specific step control function or moved continuously according to a triangular waveform. In addition to these approaches, solutions for inertia-free refocusing of the imaged plane have been developed to minimize the degree of mechanical movement. The integration of a dedicated path including AODs, such as the one described above for the XY scanning, allows for the commutation of the imaging plane within a few tenths of a microsecond. In this case, the acoustic wave encodes a frequency chirping to modulate the beam convergence properties before entering the objective [84,85,86].

Practically, such design includes two AODs driven with two counter-propagating waves to render a symmetric curvature of the light wave front. Other inertia-free approaches are based on active control of the light wave front properties based on the use of Spatial Light Modulators (SLMs) or Deformable Mirrors (DMs) [87,88].

These devices, conjugated with the back aperture of the objective, are used to impose a Zernike defocus correction pattern, in this way shifting longitudinally the focal position. From the implementation point of view, these solutions require the design of a dedicated optical layout, but do offer more flexibility in the control of the excitation beam as, along with the control of the focal position, one could impose other Zernike patterns to correct for coarse system aberrations. Whereas all the methods described above take advantage of the small size of the excitation spot to acquire the image from a densely labelled preparation, under certain circumstances, when the labelling is sparser or the detail size can be relaxed, it is possible to maximize the sampling capabilities in terms of speed by shaping laterally or axially and elongating the excitation spot. 

#### 2.2.3. Methods for Extending the Imaging Wider and Deeper in the Brain

While two-photon microscopy is a powerful tool for high resolution explorations of the neuronal circuits in limited regions of the dorsal structures of the brain, the extension of the investigation in a deeper or larger fashion faces a series of limitations. First, at increasing depths within the tissue, the light scattering tends to reduce the amount of collected photons to keep consistent the ballistic photon density of the excitation and the signal level, which necessarily requires increasing the laser intensity. Above a certain threshold increasing the light dose could more critically affect the preservation of the original properties of the sample [89,90,91]. Moreover, the SNR in MPM depend on the relative component of the fluorescence originated at the focal point with respect to the one excited in the planes out of focus. Progressively increasing the power with the depth leads to a progressive increase in the background component, ultimately limiting the signal detection capability. The effective reachable depth strongly depends on the tissue scattering properties and on the degree of labelling. In conditions of sparse labelling, it has been shown that 2PM can record from deep cortical layers (V/VI) [92]. Despite these limitations, functional imaging in deeper structures and nuclei of the brain is possible under certain conditions with a couple of methods. For high resolution imaging, a few groups proposed an experimental approach based on three-photon absorption [93,94]. In this method, typically using longer excitation wavelengths in a range between 1200–1600 nm, the impact of scattering becomes slightly less critical and the adoption of a laser source providing higher pulse energy ensures that a sufficient number of ballistic photons reach the targeted depth. In this case, to limit the average power transferred to the sample, sources with a substantially lower repetition rate are employed together with a dedicated signal detection chain. This kind of fluorescence excitation mechanism, with respect to 2PM, benefits from an increased spatial confinement, helping to reduce the level of background signal generated. Using such strategies, it has been shown that one can get clear signals from the hippocampal formation laying at about 1600 μm below the dura mater of an intact brain. Extending the functional recordings further into the depth of the brain is currently achievable only by means of miniaturized lenses that are inserted through the cortical layers and fastened to the skull [95,96]. These micro-objectives, typically between 0.3 mm and 1 mm in diameter, work as an additional component in combination with traditional confocal or multiphoton imaging systems, relying on the focal plane of the original objectives to the front of the miniaturized endoscope (“miniscopes”) and extending the effective working distance of the optical system. 

In line with this design, Gradient-Index (GRIN) optical components were successfully applied as relay elements for deep brain imaging in several flavors. GRIN optics can be customized in terms of diameter and length in order to limit the footprint and invasiveness when implanted in the animal model while preserving excellent optical properties. Typically, a GRIN rod is mechanically fixed to the skull of the animal and descends toward the target region without the need for a large tissue removal. The optical path of the acquisition and excitation system is then aligned to the GRIN lens, granting a good coupling between the microscope’s imaging plane and the distal part of the GRIN rod where a new imaging plane is formed. Contrary to fiber photometry, in which only a coarse measure of bulk fluorescence collected from the fiber tip is possible, using GRIN lenses makes it possible to reconstruct an actual image of the structures in the focal plane and, depending on the excitation/acquisition set-up, makes it possible to record functional signals with subcellular and millisecond precision. In a paper from Tang et al. [97], single-photon voltage imaging was implemented using such a GRIN-based approach in order to record in mice, whisker deflection-evoked activity in the barreloids of the ventral posteromedial nucleus of the thalamus located more than 3 mm below the cortical surface. Using this approach, it was possible to map simple and complex sensory-evoked responses to individual thalamic receptive fields while imaging a FOV of approximately 0.9 mm, encompassing the complete barreloid structure within a millisecond using a minimally invasive 1 mm thick GRIN rod. With a similar technical approach, using instead two-photon imaging coupled with a 0.5 mm thick GRIN rod, and a model of traumatic brain injury in transgenic animals, it was possible to characterize the progression of tissue degeneration with subcellular accuracy at the level of different cellular populations expressing morphological fluorescent reporters. Importantly, with this approach it was possible to track the response of the tissue for up to 60 days, maintaining the same FOV features in focus in chronic imaging sessions on the same animals [98]. This solution, originally developed for experiments with the animal’s head restrained, has also recently been applied on freely behaving conditions. In this case, along with the micro-lens, the assembly mounted on the mouse skull envisages a micro-scanner and coupling optical elements to focus the excitation beam delivered by a hollow-core fiber and collect the fluorescence signal detected by an external detector using a bundle of optical fibers [99,100,101]. This approach shows a high resolution in the imaging of subcellular compartments such as dendritic spines. When the subcellular resolution is not a critical requirement, it becomes possible to adopt miniscopes to map the population activity at cellular resolution using a simplified design based on 1PE widefield illumination. This layout, relying on small footprint complementary metal-oxide semiconductor (CMOS) camera with large pixel matrices and light sources based on light emitting diodes combined with essential combining optics, has a rather small weight (<2 g) and enables the functional recording of cellular activity over a relatively large field of view (500 μm), potentially in combination with concurrent optogenetic stimulation protocols (Figure 1e). A recording of a defined genetic profile of the neuronal population is a type of information currently not accessible with traditional electrophysiological recordings; therefore, optically recording of the average activity of a genetically specific population of neurons expressing a functional reporter represents a valuable tool. Fiber photometry offers such a possibility to gain access to deep brain neuronal populations by means of a small implantable fiber that can deliver and collect light from a small volume of tissue while the animal is freely behaving [102,103] (Figure 1d).. The detected signal, even if does not provide spatial information, holds a high temporal resolution that is not achievable with the miniscopes or other image acquisition methods, allowing for sampling faster neuronal dynamics with minimal invasiveness from deeply genetically targeted populations. 

Reconstructing the activity from wide brain circuits frequently requires not only mapping neuronal responses at different depths within the tissue but also covering relatively large areas. Regarding this last aspect, a typical two-photon microscope can capture a field of view that is rather limited with respect to the mouse brain size (0.8–1 mm vs. 12–14 mm). To overcome this limitation, a few groups have developed different methods to extend the accessible field of view to larger size, typically 2–4 mm in diameter, and engineering dedicated optical paths in combination with novel or adapted objectives [104,105,106]. Even if these solutions, from the optical point of view, maintain a subcellular resolution across such extended fields of view, for the purposes of functional imaging it becomes critical to come to a compromise between the number of the sampled points and the minimal sampling rate dictated by the kinetics of the activity reporter, typically by selecting subregions of the field of view from where to reconstruct the activity. As an alternative, when the spatially extended information is critical but the spatial resolution is no longer constrained, it is possible to reconstruct the brain wide dynamics, leveraging the advantages of optical configurations called mesoscopes (Figure 1c). In mesoscale imaging, fluorescence is excited with a brain-wide illumination with visible light and the collection of the signal with a CMOS camera from the brain surface (deeper structures are inaccessible to the visible light excitation). This approach allows for the collection of functional data from different brain regions simultaneously, with a typical spatial resolution of 60–80 μm and with a temporal resolution that is not limited by the maximal refresh rate of the camera but that is rather depending on the SNR. Indeed, this approach can capture the relatively slow dynamics of the calcium-based activity reporters (4–15 fps), the faster dynamics associated with signal of neurotransmitter reporters (50–200 fps) and the fastest signals originating from membrane voltage sensors (200–1000 fps) [107,108,109,110].

In parallel, one could consider fluorescence laminar optical tomography (FLOT) [111]. In this technique, a laser beam with a line illumination profile is scanned on the sample and the emission signal is captured by a sensitive camera. Image reconstruction algorithms are used to retrieve the original signal from different depths.

Axial resolution of around 200 μm can be achieved for several millimeters. Interestingly, this technique can be used to perform time-resolved imaging, for example, in the perfused rat heart loaded with voltage sensitive dyes and to reconstruct the 3D shape and dynamics of its electrical activity [112]. With appropriate modification of the acquisition setup, it is possible to increase both resolution and depth sensitivity. For example, angle FLOT (aFLOT) was applied to voltage imaging of the barrel cortex in mice receiving single-whisker tactile stimuli. As a result, the 3D electrical activity reported by the voltage sensitive dye could be reconstructed with a 200 Hz sampling rate, enabling the volumetric mapping of the propagation of a sensory-evoked neuronal response across the whole barrel cortex thickness in a mesoscopic depth-resolved acquisition system [113].

## 3. Molecular and Hardware Tools to Modulate Brain Activity

Measuring the neuronal activity, either from a specific region or across the whole brain, represents an invaluable tool to pinpoint extended or localized alterations of the circuit mechanisms associated with a pathological condition. Such identification of the altered dynamics is fundamental to eventually bring the investigators closer to the determination of the causes and of the origins of the identified abnormalities. However, this approach is not capable, per se, of revealing to what extent certain neuronal components are required to support processing or to clearly dissect the origin of the abnormal activity recorded. This is because reconstructing the activity of a neuronal circuit provides mostly a correlative type of information, i.e., one event occurs concurrently with another, not offering the possibility to unravel causality relationships between the ongoing dynamics. Furthermore, even if the origin of the abnormalities could be identified, exploiting that information in the translational perspective would easily require a method to intervene on the system with a spatial-, temporal- and cell-specific manner according to the information available. With respect to activity recording approaches, neuromodulation methods could allow for precisely evaluating the impact of a specific population on the circuit dynamics on one side and, on the other side, for re-tuning the circuits closer to the physiological functionality, at least at the level of a proof-of-principle on a model organism of the disease.

Traditionally, the modulation of circuit activity has been performed with electrophysiological and chemical approaches. These methods, independent of the level of invasiveness, typically miss the required specificity and flexibility to bring their effects to the identified neuronal populations or cellular compartments, and come with a number of side-effects, sometimes masking or altering the observable outcomes. In recent decades, on one hand, the techniques of molecular biology have offered unprecedented capabilities for the engineering of molecules and their targeting to specific cellular populations or compartments. On the other hand, new optical technologies have enabled researchers to work with model organisms with minimal invasiveness more frequently each day in conscious and freely behaving conditions. This has led in the last 10 years to the refinement of genetically encoded light-activated molecules capable of interacting with the targeted cellular signaling in general and to bi-directionally modulate the membrane potential in specific neuronal sub-populations. These tools bring together the advantages of the spatio-temporal precise light delivery in the living tissue with the genetic specificity allowed by the techniques of molecular engineering, prompting the emergence of a completely new field called optogenetics. From the original idea of using light to actively operate on the system, the concept has been extended to also cover light-based approaches relying on genetically encoded molecules to record neuronal activity. 

In order to get the most out of these tools, many labs have contributed to the development of optical technologies capable of supporting the researchers in the investigation of the neuronal circuits. Following the first approaches, where the activity of populations of neurons was modulated by using an optic fiber to shine the light over a targeted region, more sophisticated methods have been refined to increase the spatial precision of photostimulation. These latter methods, relying on the illumination of the sample with an arbitrary three-dimensional distribution of light, allow for high spatial and temporal precision in the modulation of arbitrary groups of neurons with cellular resolution across different compartments of a neuronal circuit. Since 2014, this high-resolution light-based modulation has been integrated with optical recordings of the neuronal activity, resulting in the creation of “all-optical” protocols, allowing for probing of neuronal circuits at cellular resolution [114,115,116]. 

In the following paragraphs, we will first describe the currently available toolbox of light-based modulators of neuronal activity, focusing on those genetically encoded and their basic working mechanisms. Then, we will report on the optical techniques currently available to use these tools in the investigation of neuronal mechanisms in physiological and pathological conditions. 

### 3.1. Molecular Tools to Dissect Brain Activity

#### 3.1.1. Synthetic Tools to Dissect Brain Activity

The large family of light-based modulators of neuronal activity is generally divided into two main branches, the class of synthetic compounds and the class of genetically encoded molecules. Synthetic optogenetics traditionally represents the first approach using light to control cellular processes and is currently an umbrella term covering a variety of tools and techniques, such as chemical-optogenetics, photo-pharmacology or optogenetic pharmacology. Their general design envisages conferring light-sensitivity to a protein or a molecule. In contrast to the second class of genetically encoded optogenetic tools that incorporate naturally occurring chromophores, this strategy employs synthetic, light-sensitive photoswitches that undergo chemical reactions following the absorption of specific wavelengths. One of the most common mechanisms relies on the trans-to-cis isomerization of azobenzene, resulting in a large geometrical change and significant reduction in the length of the molecule. Azobenzenes are mostly in –trans configuration in the dark or following green-light illumination, but isomerize to the –cis form upon UV light absorption. Therefore, isomerization can be utilized to alter/modulate a protein’s and molecule’s functionality. Different strategies of conjugation of these moieties with intrinsic molecules and proteins, or exogenous molecules, have produced a large library of photo-uncaged compounds such as allosteric modulators of receptors [117,118,119,120] and photo-switchable channels [121,122,123,124,125] which are an invaluable tool for probing cellular mechanisms. On the other hand, we find the class of the genetically encoded light-based modulators of the neuronal activity. 

#### 3.1.2. Genetic Tools to Optically Dissect Brain Activity

**Figure 2:**
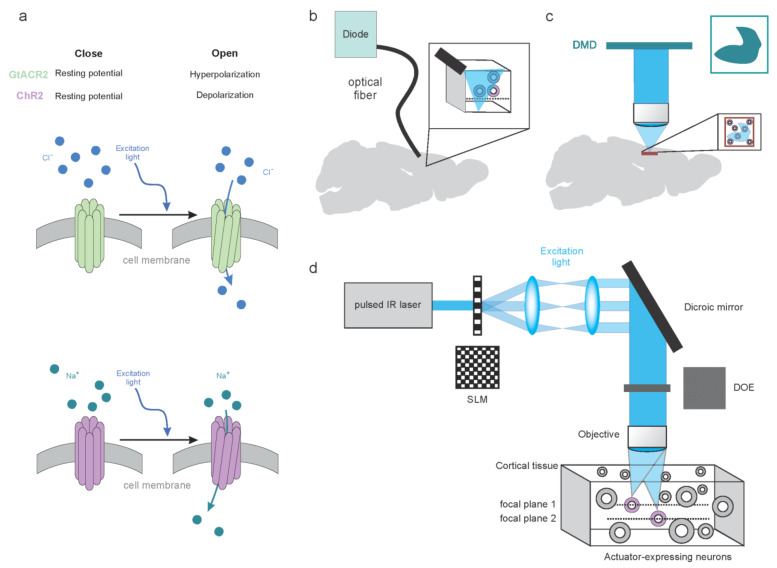
Optical methods to modulate neuronal activity. (**a**) Example of light-gated channels. Upon illumination, the channels open and allow the movement of ions across the membrane based on their selectivity. (**b**) In the simplest configuration, excitation light can be delivered by an optic fiber placed near the target area. (**c**) A more precise control of the illumination can be obtained relying on DMDs which create pattern of light based on the on–off configuration of the individual micromirror. (**d**) Spatial light modulators further stress the control of illumination patterns by extending it to the three dimensions.

Nowadays, modulation of neurons and their activity mostly takes advantage of a large toolbox of genetically encoded light-sensitive channels and pumps. These membrane proteins (termed opsins) are typically derived from structures found in animal mechanisms of light conversion and they all rely on a natural chromophore in the form of retinal bound to a portion of these molecules. When retinal absorbs a photon, an isomerization process is activated, resulting in a series of conformational changes favoring, with characteristic temporal dynamics, the diffusion or the transportation of ions across the membrane (Figure 2a). This translocation of charged ions is the mechanism underlying the light-induced modulation of the membrane potential and, consequently, the probability of favoring or inhibiting (according to the type of opsin) action potential firing. The specificity in ion permeability of these channels or pump results in either a depolarization or a hyperpolarization of the neuronal cell. Along with their ion specificity, these molecules come with different features in terms of spectral sensitivity, ion conductance and temporal dynamics (Table 2).

Depolarizing opsins drive inward currents by allowing the movement of cations and protons inside the cells. Channelrhodopsin-2 (ChR2) has been the first-used optogenetic tool; it is a blue light-activated cation channel derived from a green alga [136]. Since its discovery, it has been used as a backbone for the development of variants to enhance specific features such as spectral sensitivity, photocycle kinetics and conductance [137,138]. Keeping track of neuronal activity during light-induced modulation should be favorable. However, the most commonly used calcium reporters, such as the GFP-derived ones, have sensitivity in the blue spectrum, so the use of opsins with separated action spectra is essential to prevent cross-talking between the two molecules [11,130,139]. This led to the generation of variants with red-shifted action spectra, among them Chrimson, with its faster mutants [127,131], and ChRmine [130]. To favor temporally precise manipulation, photocycle kinetics are of crucial importance. Indeed, opsins with fast closing rates, or tau off, allow for sustained action potential spiking rates in response to a long train of impulses and prevent refractoriness of the neuron [140]. However, the enhanced photocycle kinetic comes at the cost of a decreased light sensitivity, meaning that high powers of irradiance are required, potentially damaging the tissue either by heating or phototoxicity. Thus, the generation of opsins combining fast kinetics and high-light sensitivity has been preferred. The wild-type ChR2 has a tau on of 1.7 ± 0.1 ms and tau off of 10 ± 0.8 ms and it can generate spikes at an irradiance level of ~1 mW/mm^2^ [126]. Chronos (ShChR), a mutated form of ChR, has a tau on of around 2.3 ms and a tau off of 3.6 ms, making it one of the fastest opsins, and reliably driving neuronal spiking at low-light power, 0.05 mW/mm^2^ [127]. From Chronos, a new opsin has been derived, ChroME [128]; the two molecules share similar action spectra and kinetics, but the latter displays greater photocurrent amplitude; hence, lower laser power and shorter light pulses are required to elicit spikes. Recently, ChroME has been used as a scaffold for the generation of new variants, namely ChroME2f and ChroMe2s, with enhanced photocurrent and fast decay time, respectively [129].

Hyperpolarizing opsins drive outward currents, mediating either the influx of anions via light-gated channels and pumps or the efflux of protons. Silencing of the neurons during an experiment can either require opsins with fast kinetics that allow precise temporal control, such as during behavioral experiments, or opsins with slow kinetics that suppress the action potential initiation for the entire duration of the experiment. A widely used hyperpolarizing opsin is Halorodopsin (eNpHR3.0), a yellow light-driven ion pump selective for Cl^−^ that combines larger photocurrents driven by low light power (3.5 mW/mm^2^) with fast kinetics in the millisecond range [134,141]. eArch3.0 is a yellow light-driven proton pump with fast kinetics (tau off ~9 ms) [133]. However, this pump can induce intracellular pH changes, leading to Ca^2+^ influx at the presynaptic level and then to spontaneous vesicular release [139]. In 2015, two light-gated anion channels were isolated from a cryptophyte (GtACR1, GtACR2) [132]. They both present a high selectivity for chloride, high conductivity and can efficiently suppress spiking at low irradiance, 0.005 mW/mm^2^. The kinetics, as well as the spectra sensitivity, are different between the two proteins with GtACR2 being faster (0.04 s) and more green-shifted. The identification of these new anion channels, together with the production of engineered opsins [142,143], has catalyzed the development of new silencing tools, leveraging the greater conductivity and photocurrent stability provided by the channels [135,144,145,146,147]. Every opsin has limitations and in general, efforts are required to carefully select the most appropriate molecule. For instance, neuronal inhibition in live animals should require hyperpolarizing opsins with fast kinetics and low irradiance such as Halorodopsin, which, however, is not suitable when the amplitude and duration of the stimulation are higher, as this leads to alteration in the network excitability [148]; in this case, GtACRs or Arch are more suited. The effect of channel opsins depends on the electrochemical gradient which can change during neuronal development and across compartments. For instance, GtACRs will drive an inward current, depolarizing the cell, if expressed in immature neurons or neuronal compartments with a high intracellular chloride concentration [149]. 

### 3.2. Optical Approaches to Dissect Brain Activity

Using light sensitive tools to control the membrane potential or more, in general, the cell signalling assumes that optical methods are in place to deliver light into the sample according to an appropriate spatio-temporal pattern derived from experimental questions. The major constrains for this task come from the absorption of the light into the tissue, limiting the depth within the tissue where light can be delivered, and the size and position of the population one would like to target with the modulation. Within these tenets, current technologies allow for a range of modulation scenarios, from the activation of large areas of the brain at once to the cell resolution control of the activity in hundreds of identified cells in three dimensions. There is no optical approach that can currently cover both these scenarios, so the subsequent paragraphs will focus on summarizing the options available for the different conditions.

#### 3.2.1. Low-Resolution Modulation of the Neuronal Circuits

Even though there is no clear definition for low-resolution neuromodulation strategies and the boundaries of these approaches can vary from case to case, this concept includes all the techniques allowing for addressing light in the visible range of the spectrum and, consequently, the modulation, in a defined region of the brain. The size of the region targeted can be as small as a patch of 50 μm in diameter or as big as a brain cortical area or more. In this case, the goal is typically to recruit a subpopulation of neurons that are prominent in one region. This approach is also known as “widefield” stimulation, to highlight the fact that all the opsin expressing cells belonging to the illuminated region are recruited at the same time. The techniques adopted for this scheme in general can be very different. A common microscope objective can be used to deliver into the sample the visible excitation light generated by a fluorescent lamp or a light-emitting diode, and by reducing the size of the microscope field aperture, the effective stimulated region is limited. Selection of the region of stimulation requires, in this case, moving either the objective or the sample in proximity to the other, while the temporal protocol can be easily supported by an electronically actuated shutter under the control of a digital signal. Even if this is a very effective method perfectly working in combination with electrophysiological recording in dissociated cultures or ex-vivo slices, traditionally, most of the neurostimulation studies have used a light delivery approach based on optic fibers (Figure 2b). Light generated by a source, typically a laser or a light-emitting diode is coupled in at one end of the fiber and the light is delivered by this flexible guide to the other end of fiber, where it exits with an illumination cone whose divergence depends on the numerical aperture of the fiber. Similar to fiber-photometry applications, with this approach, light can be delivered at meters of distance from the source and be easily targeted in regions different from the area of observation. The peculiar advantage of this photo-stimulation approach is that by gluing or cementing the output end of the fiber to the skull of the animal, one could potentially modulate the neuronal activity when the animal is engaged in the behavioral activity. The fiber-based strategy has been adopted in several studies targeting populations of neurons in the superficial structures of the brain, such as cortical areas, but it has also been used for targeting structures deep in the brain, such as the amygdala and hypothalamus, by inserting the fiber in a small-diameter canula cemented into the skull and reaching the targeted region. Targeting the neuromodulation on multiple regions with the methods mentioned above is in general not possible or requires a complicated design of bundles of optical fibers where each fiber is driven to illuminate a different area of the brain. This represents the first drawback of these approaches, that is the lack of the possibility to target the neuronal modulation according to arbitrary spatial stimulation patterns, designed with multiple regions of different shape and size. To overcome these limitations, many groups have taken advantage of digital micromirror devices (DMDs, Figure 2c). These are microelectronics systems that are commonly found in the video projectors and are based on a large matrix of small mirrors. Basically, when these systems are inserted along the microscope optical path and properly conjugated, the excitation light from the source is reflected by the surface of the mirrors on the sample and turning a mirror from OFF to ON position corresponds by illuminating the corresponding spot at the sample plane. This method allows for structuring the photo-stimulation spatial profile in the two dimensions orthogonally to the light propagation direction according to the regions of interest. Moreover, having these devices typically refreshes times ranging from microseconds to milliseconds, and allows for extremely fast switching between different stimulation patterns. Even if with the DMDs one could, with a proper design of the optical system, structure the photo stimulation pattern and achieve, in theory, a resolution close to single neuronal cells, there are other aspects that one has to consider and that renders this approach suitable only for low resolution neuromodulation. First, the laws of light diffraction dictate that there is a linear relation between the lateral size of the illuminated patch and its extension along the light propagation direction, so that, when considering planes out-of-focus, the probability of excited neuronal cell remains relevant compared to in-focus planes, limiting, in a substantial manner, the 3D resolution. Second, partially because of the previous point and because of the optical design, there is no possibility to selectively target the neuromodulation to different structures displaced in the 3D space, at least without considering the engineering of more complex optical configurations. Third, DMDs work by splitting the total input energy across the matrix surface and this means that the stimulation power density at the sample cannot arbitrary increase but is bound to the maximal value achievable depending on the total area of illumination. A critical aspect to consider when dealing with the resolution in the modulation is that the expression of the opsins is, in general, not restricted or only partially concentrated at the level of the soma. Dendritic arborization and axonal projections form a dense mesh surrounding the cell bodies of other cells potentially located far away from the originating cells. These passing-by network of processes are a factor limiting the achievable spatial resolution and the interpretation of the experimental outcomes. To overcome these aspects and limit the spurious activation of the neuronal substrates, different approaches have been developed, and these will be described in the next paragraph. 

#### 3.2.2. High-Resolution Modulation of the Neuronal Circuits

Optical methods for modulating the activity at high resolution typically refers to a series of approaches where light distribution can be targeted or engineered in such a way to specifically address one or more cells without altering the activity of nearby units. Common fundamental elements for these approaches is that the opsins are activated via multiphoton; in fact, most of the opsins present a relatively large excitation cross-section in the wavelengths between 900–1100 nm, assuring that these molecules can be excited with two-photon excitation. Moreover, using two-photon excitation is fundamental to confine the excitation along the longitudinal dimension, and this basically is derived from two aspects. First, as mentioned above, the law of diffraction dictates a relationship between the lateral and the longitudinal size of the excitation spot; the larger the spot, the more extended its tails are along the z-axis. However, whereas in the case of linear absorption light intensity decays following a 1/r^2^curve, where r is the longitudinal distance from the focus, in the case of non-linear two-photon excitation, absorption probability away from the focus decays more rapidly, with a 1/r^4^ relation. Second, a further improvement of the longitudinal confinement of the excitation spots can be achieved considering the fact that multiphoton excitation uses laser sources emitting short pulses of light, usually in the order of hundreds of femtoseconds. In this case, a technique of controlled temporal dispersion of the light pulses called “temporal focusing” can be implemented, so that the absorption probability, depending linearly on the inverse of the duration of light pulse, is enhanced in the focal plane and reduced in the region out of focus. This combination allows for confining the opsin excitation at the plane corresponding to the focal position and preserves the spatial resolution in three dimensions. Independent of the particular scheme and hardware configuration, it is important to keep in mind two important facts: that the single channel conductance of these opsins is generally extremely small, well below the values corresponding to many common voltage-gated ion channels involved in the generation of action potentials; and that the opsin density on the membrane surface might vary from cell to cell and from an experimental condition to the other. It follows then that for an effective modulation, one has to maximize the ion transport across the membrane. This is typically achieved, leveraging a mechanism of spatio-temporal integration of the photocurrent, either by quickly scanning the beam along a spiral trajectory across the somatic membrane (“scanning approach”) or, alternatively, illuminating the cell body with a 2D light patch of the corresponding size (“scanless approach”). Whereas the first approach is rather straight-forward, as it requires the programming of the microscope XY scanner to follow a defined trajectory, the scanless method envisages the use of additional optical elements to correct the beam propagation properties upstream of the objective to extend the lateral width of the excitation spot. In this case, one can opt either for a static optical element, such as a beam expander, to limit the beam diameter and obtain an extension of the focal spot, or a dynamic component capable of quickly and easily re-configuring the light wavefront entering the objective in a way to change the size of the illuminated spot at the sample according to the requirements. Dynamic control of the light wavefront is typically obtained with devices called Spatial Light Modulators (SLMs), which are large matrixes of liquid crystals where the refractive index of each pixel can be individually controlled with an electric signal, with the result of delaying the light wavefront in that position of an amount proportional to the amplitude of the control signal. According to optics law, there is a mathematical relationship linking the light intensity distribution at the sample to the geometrical features of the wavefront; therefore, it is possible to engineer the shape and size of the illuminated areas by properly configuring the map of delays on the SLMs (Figure 2d). 

## 4. Light-Based Brain Circuit Analysis and Modulation in Pathological Conditions

Functional imaging of large populations of neurons along with targeted modulation of distinct cell subtypes have proven to be invaluable tools to dissect the mechanisms underlying neurological and psychiatric disorders. Evidence from clinical and preclinical studies indicates that coordinated activity among groups of neurons within local and wider networks are impaired in the early stages of several neurological and neuropsychiatric disorders. These aberrant rhythms evolve and change along the progression of the disease, representing a signature of distinct pathological stages. Although this trait emerges as a common feature, how specific cellular, molecular and circuit alterations impact network dynamics in different disorders remain unknown, leaving unaddressed how distinct defects connect to a coherent pathophysiological scenario. However, recent studies have provided important results in that direction.

### 4.1. Neuropsychiatric and Neurological Disorders

#### Schizophrenia

Schizophrenia (SCZ) is complex disorder which originates from a combination of genetic, epigenetic and environmental factors. In the past several decades, the understanding of the mechanism underlying psychiatric disorders has progressed significantly. In humans, alterations in excitatory/inhibitory balance, neurodevelopmental processes and neuromodulation have been identified at cellular, molecular and synaptic level [150]. At the systems level, alterations in functional connectivity and synchrony, resulting in aberrant gamma oscillations in cognitive and sensory brain areas, have been shown to be present since the early onset of the disorder [151,152]. The neuronal circuits underlying such alterations remain largely to be identified, and a clear etiopathogenetic mechanism underlying SCZ is still missing. Much computational [153] and experimental evidence seems to indicate that the core of SCZ pathology could be related to diminished stability and altered dynamics of neuronal circuits supporting cognitive and sensory processing. In particular, as mounting evidence indicates that coactive groups of neurons (ensembles) represent the “building blocks of functional circuits” [154,155], an emerging vision postulates that the breakdown of coordination within these neuronal ensembles could represent the key feature in the etiopathogenesis of SCZ. Recent studies investigated neuronal activity using large-scale functional imaging to track neuronal activity in the primary visual cortex (V1) in established mouse models of SCZ [156,157]. They found that single-cell properties, such as the average activity of individual neurons, and orientation/direction selectivity, were unperturbed in Setd1^−/−^ mouse model of SCZ. However, coordinated activity within ensembles of neurons in V1 was deeply altered both at rest and in response to sensory stimulation. According to the role of coordinated activity among groups of neurons in coding sensory stimuli [154,158], in control mice, sensory stimuli elicited highly correlated activity in orientation/direction responsive cells. In contrast, in mutant mice, although visual stimuli elicited a prompt response, patterns of correlated activity were compromised [157]. Similar results were obtained in Df (16) A^+/−^ modeling 22q11 microdeletions [156]. Again, individual cell response properties in V1 were not altered; however, functional imaging of large population of cells revealed impairments in correlated activity among an ensemble of neurons in V1. Altogether, these results posit that the different genetic, cellular and molecular risk-signaling pathways associated with SCZ may converge into a common mechanism at population level, disrupting the correlated activity within ensembles of neurons. This breakdown of coordinated activity could affect neuronal dynamics within local and wider networks in different brain regions, resulting in impaired sensory and cognitive functions. Even though alterations in gamma and alpha oscillations were known to be present both in patients and mouse models of the disease, performing large- scale functional imaging in mouse models revealed the cellular elements involved in such alterations, and the specific defects underlying the identified alterations. These results, as important as they are to understanding the pathophysiology of SCZ, also have the potential to pave the path for new therapeutic approaches based on neuromodulations of specific targets within the disrupted ensembles, and to restore the physiological activity and hence rescue disrupted functions. To this end, evidence indicates how ensembles of neurons, present both at rest and in response to sensory stimulations in V1, can also be imprinted de novo, by repetitive optical stimulations of distinct subsets of neurons. Once imprinted, these co-active groups can be recalled by stimulating just one or a few elements of the ensemble, proving that correlated activity among neurons strengthen their synaptic connections, according to the Hebbian rule [155]. More recently, in a novel model of SCZ, hyperactivation of cortical neurons was observed during resting states (i.e., in the absence of sensory stimulation) as a consequence of an elevated frequency of spontaneous neuronal firing. These observations were linked to increased presynaptic activity, providing a possible explanation for visual hallucinations in SCZ [159]. On a similar line of evidence, two-photon calcium imaging was used to record visual cortex activity in wild-type animals during hallucinogenic episodes triggered by exogenous serotonin agonist administration. The altered pattern of activity was related to a reduction in sensory-evoked activity, cell-type specific alteration of temporal processing and a compromised surround-suppression processing, supporting a bottom-up perspective in SCZ [160]. Calcium imaging was used to test the effect of therapeutic approaches in a mouse model of SCZ, demonstrating the potentiality of drug screening in such contexts. Briefly, the aberrant network activity in medial prefrontal cortex (mPFC), as recorded after pro-psychotic treatment, was effectively restored to control levels after administration of antidepressants and Metformin [161]. Modulatory optogenetic approaches aimed at controlling local and global network activity in SCZ models, recently showed interesting potential in restoring functional properties close to healthy conditions in SCZ mouse models. In a recent paper, constant optogenetic stimulation of the basal forebrain ascending arousal system was demonstrated to be efficient in controlling broad gamma band power replicating symptoms reminiscent of human SCZ, whereas tonic inhibition reduced the ketamine-induced gamma power elevation also present in SCZ [162]. Furthermore, hyperactivity in the human anterior hippocampus has been identified as a potential predictive marker for SCZ. Optogenetic activation of the ventral hippocampus evoked SCZ-like symptoms in wild-type mice [163]. Similarly, replicating the delta frequency increase observed in thalamic nucleus reuniens with optogenetic stimulation triggered working memory malfunctions in otherwise normal mice, further supporting the evidence that network level disruption of intrinsic rythmogenesis is a hallmark of SCZ [164].

Altogether, these findings shift the focus of the research at the neuronal circuits level, considered to be the critical architecture for processing neuronal information, and whose disruption may lead to psychiatric disorders. These results also indicated that therapeutic approaches for SCZ, as well as other psychiatric disorders, must aim at restoring neuronal activity at the systems level, to reintegrate the selective cooperation of distinct groups of neurons which represent the functional unit of information processing in the brain.

### 4.2. Alzheimer Disorder 

As mentioned above, aberrant oscillations are a signature not only of neuropsychiatric but also of neurodegenerative disorders [165]. In Alzheimer Disease (AD), gamma oscillations were reported to be reduced in amplitude during the early stage of the disease, both in humans and in mouse models with AD [166,167]. The interplay between this altered brain rhythm and the pathology remains poorly understood. In general, it is known that cellular and molecular pathology affects first the synaptic functionality. In AD, it has also been shown the inverted relation, such that increased synaptic activity, favors the production of Aβ, which is known to exert toxic effects, resulting in inflammation, synaptic alterations and culminating in neuronal loss [168,169]. Entraining gamma oscillations in a mouse model of AD (5XFAD) could have an impact on AD pathology. They found that optogenetic modulation of hippocampal parvalbumin (PV) interneurons at gamma frequency (40 Hz), but not at other frequencies, reduced the load of amyloid Aβ (Aβ 1–40 and Aβ 1–41 isoforms). Furthermore, they found that this optical stimulation induced a change in the morphology of microglial cells that presented thicker processes and bigger soma. These changes in cell morphology indicate an increased phagocytosis, as confirmed by a higher load of Aβ into microglial cells, suggesting their involvement in reducing the extracellular load of Aβ. Thus, entraining gamma oscillations in the hippocampus appears to have an overall protective effect, both on neuronal and on microglial cells. Subsequent studies revealed that entraining gamma oscillations exert protective effects on neurons and synaptic density in several brain areas [170]. These results, although important to understanding the etiopathogenesis of AD, are also relevant to design new therapeutic approaches aimed at exploiting targeted neuromodulation to exert a protective effect against AD pathology [168]. Furthermore, these observations prompt speculation that this intervention could also impact the evolution of the disorder, slowing down or even halting its progression.

### 4.3. Parkinson’s Disease

Parkinson’s disease (PD), the second most common progressive neurodegenerative disorder, is characterized by distinct motor impairments due to the accumulation in the basal ganglia of misfolded, phosphorylated proteins, mostly composed of alfa synuclein (alfa-syn). Prior to motor impairments, PD patients exhibit several non-motor symptoms which appear during a long prodromal phase. Among these, olfactory deficits, present in more than 90% of PD patients, are among the earliest ones and can precede the onset of motor impairments by years. The progression of the clinical symptoms reflects changes in the pathology in the central nervous system (CNS). Imaging and neuropathological evidence along with clinical and epidemiological data indicate that the olfactory bulb, the first brain area where olfactory information is elaborated, is one of the first regions of the CNS where aggregates of pathogenic proteins accumulate [171,172]. From here, pathologic alfa-syn spreads to higher brain areas following stereotypical pathways. The spreading capability of pathologic alfa-syn has also been demonstrated in a mouse model of PD by injecting preformed alfa-syn fibrils in the OB and following their progression across the brain at different time points. This approach revealed that the injected alfa-syn fibrils recruits the endogenous alfa-syn in pathological aggregates that spread transneuronally at first in the olfactory areas and then in more distant, non-olfactory brain regions over several months [173]. Despite the high prevalence, the mechanisms underpinning olfactory deficits remains unknown. In addition, even though alfa syn is a presynaptic protein [174,175] and most of the genes associated to familiar PD encode proteins associated to the synapse [176], the impact of these proteins on neuronal circuit activity, at the systems level, has remained elusive for long time. Recently, two studies addressed these critical open questions. To pinpoint the impact of genes associated to PD on olfaction, Maset et al. [177] investigated the role of LRRK2, the most common cause of familiar PD, on olfactory processing. They found that at the behavioral level, LRRK2 KO mice exhibit olfactory deficits. Whereas functional imaging in the olfactory bulb unraveled, odor stimulation mutant neurons have smaller, less reliable responses and exhibit a spatio-temporal profile with reduced correlation [177]. Regarding the role of alfa-syn fibrils in the sensory cortex [178], performed functional imaging in the somatosensory cortex (SS1) of a mouse model with PD. Imaging was performed in conscious mice 9 months after intra-striatal infusion of preformed alfa-syn fibrils. Cortical neurons in layers 2/3 exhibited deeply altered responses in the mouse model of PD with respect to controls. The fraction of responsive neurons to whisking was increased and each neuron presented larger stimulus-evoked responses than in WT controls. Furthermore, during whisking, neuronal activity appeared more correlated in alfa-syn PD model mice than in WT controls [178].

Deep Brain Stimulation (DBS) is a therapeutic intervention applied to intractable neurological and psychiatric disorders. In PD, high frequency stimulation (HFS) of the subthalamic nucleus (STN) has provided an effective intervention for medically non-responsive PD [179,180]. Although promising, the effects of DBS in PD are partial and limited in time. One of the major limitations of this approach is that the mechanisms through which DBS exerts its therapeutic effect are unknown, for several reasons. First, the target area is characterized by a significant heterogeneity of the tissue, such that it is not known on which neuronal elements DBS acts. Second, the pattern of the stimulation is pretty coarse. HFS is a complex manipulation that can elicit increase or decrease of neuronal activity patterns or even a mix of the two patterns in the target neurons. Last, electrical stimulation produces artefacts, preventing the understanding of the outcome of the stimulation at circuits level [181]. In this scenario, optogenetics stimulation/inhibition of distinct components of neuronal circuits in animal models of diseases proved to be the ideal approach for dissecting the different elements of the pathological circuits and identifying targets for effective interventions. Aiming at understanding the mechanisms underlying DBS and improving its efficacy, optical stimulation was applied to a pharmacological model of PD in rodents [182]. Optical inhibition of STN was found to have little therapeutic effect on the impaired movements of Parkinsonian rodents. Similar results were obtained by Yoon et al. [183] who performed optical inhibition of STN and observed a partial recovery of motor impairments in a rat PD model. In contrast, selective optical stimulation of axonal projections to the STN potently reduces STN neuron spiking and restores the motor behavior to normal level. Interestingly, low frequency stimulation (LFS) of the same afferents as the SNT, exerted the opposite effects, worsening the motor impairment in Parkinsonian rats. Furthermore, it was found that the HFS of layer V neurons in the primary motor cortex was able to rescue the motor behavior in PD rats, whereas again LFS worsened the pathological symptoms. All together, these results show the power of optogenetic stimulation in dissecting the pathological circuits and identifying selective target and proper temporal patterns of stimulation to accomplish effective therapeutic interventions [182]. Selectivity of the target and specific frequency and pattern of stimulation proved to be critical to also exert a positive effect in a different paradigm. Seeger-Armbruster and colleagues [184] found that theta burst, but not tonic stimulation of the glutamatergic fibers in the motor thalamus, significantly improved reaching in Parkinsonian rats. Although most studies have focused on stimulation of deep brain structures to ameliorate PD symptoms, this approach is invasive and benefits a limited number of PD patients due to the stringent criteria of eligibility. To test alternative approaches for controlling PD symptoms, a more recent study found that the optical drive of the secondary motor cortex (M2) improves motor symptoms in a dopamine-depleted mouse model [185]. Glutamatergic neurons in an M2 project to subcortical structures involved in motor control, makes synapses with dopaminergic neurons. Therefore, optical excitation of M2 neurons or projections to the dorsomedial striatum increases dopamine release and favors motor coordination. It is noteworthy that stimulation of M2 along with L-DOPA treatment improved working memory performance, which was absent in mice treated only with L-DOPA. These results reveal that M2-basal ganglia circuits are critically involved in motor and cognitive function, and demonstrate the efficacy of the optical drive of cortical glutamatergic projections from M2 to improve motor and cognitive processes. Looking at less invasive therapeutic approaches, opto-activation of cortical somatostatin interneurons was found to be effective in alleviating motor symptoms in a mouse model with PD. Furthermore, optogenetic stimulation was combined with pharmacological treatment with L-DOPA to enhance the therapeutic effects, and also to control L-DOPA-induced dyskinesia (LID). The optical drive of striatal cholinergic interneurons was found to reduce LID. Once again, the pattern of stimulation was critical to achieve the effects, as short optical pulses increase LID, whereas long optical pulses reduced LID, acting on nAChR and mAChR. These data contributed to identifying new elements in PD pathological circuits, revealing the critical role of striatal cholinergic interneurons in the genesis and control of LID and suggest that a cholinergic agonist and antagonist could contribute to limiting LID in PD [186]. 

### 4.4. Stroke

The focus of the research on stroke, i.e., an acute ischemic event in the central nervous system, is to limit the damage caused by the ischemia and favor the recovery of the neurological functions. To accomplish these goals, the research has to: 1. follow the progression of the possible alterations of neuronal network dynamics around and far from the ischemic area and 2. design appropriate neuromodulation interventions to rescue neuronal activity and functions. Mesoscopic imaging has been the gold standard to monitor blood flow and neuronal circuits dynamics in large brain areas after a stroke in several mouse models. Balbi et al. [187] established a bi-hemispheric transcranial window for longitudinal mesoscopic imaging of cortical function upon acute stroke in conscious head-fix mice. Combining laser speckle contrast imaging and wide-field calcium imaging, Balbi et al. investigated the effects of the cortical spreading ischemic depolarization induced by the stroke. In mice expressing the genetically encoded calcium sensor, an ischemic depolarization wave (3–5 mm/min) propagated across the cortex 1–5 min after the stroke. Blood flow, monitored by speckle imaging, was found to correlate with lesion volume and neurological impairments. The possibility to monitor neuronal activity soon after the stroke and longitudinally is essential to design appropriate neuromodulation interventions.

Optogenetic stimulation has been largely used in mouse models of stroke to re-establish the excitatory–inhibitory balance in neuronal networks. In a mouse model of stroke upon middle cerebral artery occlusion (MCAO), stimulation of striatal GABAergic interneurons expressing ChR2 (excitatory effects) and Arch (inhibitory effects) revealed that the inhibition of GABAergic striatal interneurons favors functional recovery. Namely, GABAergic interneurons inhibition reduces the volume of the atrophic brain area by increasing micro-vessel density and limiting cell death. On the contrary, stimulation of GABAergic striatal interneurons promoted detrimental effects that worsen the pathological situation [188]. Furthermore, it has been found that optical inhibition of striatal neurons promotes neurogenesis and favors functional recovery in MCAO stroke mouse models [189]. Optogenetic stimulation of selective neurons ipsilaterally to the stroke, in the primary motor cortex, was shown to promote function recovery. Repeated optical stimulations increase blood flow in the ischemic area and neurovascular coupling. In addition, activity dependent neurotrophin expression was higher in the stimulated area. All these events contributed to favor function recovery as also demonstrated by the better motor behavior performance of optically stimulated mice with respect to controls [190]. Optogenetic inhibition of striatal neurons was shown to also be effective in promoting survival and integration of neuronal stem cells (NSC) upon MCAO, and in reducing the volume of the ischemic area. Altogether, these events further improved neurological recovery [191].

To restore neurological function, the spared neuronal circuits have to reorganize and reactivate. This reorganization involves not only the area interested by the stroke, but also the connected areas. In particular, ischemic events in the somatosensory cortex were shown, by functional imaging, to reduce neuronal excitability not just in the ischemic cortex but also in the thalamo-cortical projections. It is noteworthy that optical stimulation of the surviving thalamo-cortical axons favors new and stable connections. Furthermore, chronic optical stimulation was found to restore somatosensory circuit function, sensory motor integration and behavioral outcome. These data demonstrated that optical stimulation of thalamic circuits favor rewiring and functional recovery [192]. In most cases, optogenetic neuromodulation was found to favor recovery of neurological function when applied weeks after the ischemic event. A recent study [193] demonstrated the beneficial effects of optogenetic entrainment in the gamma frequency one hour after the ischemic event. A photothrombotic stroke was induced in the forelimb sensory and motor cortex in mice genetically expressing ChR2. Optical stimulation at 40 Hz of the peri-ischemic zone rescued neuronal activity with 24 h from the stroke and increased blood flow over the first week after the stroke. Neuromodulation reduces the volume of the ischemic area and favors the rescue of the motor function. These results indicate that modulation of oscillatory activity in the very early phase after stroke can have significant beneficial effects and provide important suggestions to improve the efficacy of therapeutic protocols. 

### 4.5. Epilepsy

Abnormal neuronal dynamics are the pathological signature of epilepsy, a neurological disorder characterized by spontaneous and recurrent seizures that are generated by abnormal neuronal activity and hyper-synchrony in various brain areas. Seizures, according to the areas involved, cause cognitive, sensory and emotional alterations and frank deficits [194]. Current antiepileptic treatments often fail in controlling the seizures. This limitation is mostly due to the lack of spatial and temporal resolution of these pharmacological treatments, which do not target specific neurons within a precise temporal window but attempt to broadly control neuronal excitability. This approach can at best control symptoms while producing several and often severe side effects. Functional imaging of the epileptic brain areas enabled inference of the pattern and the cellular elements involved in the epileptic network. Targeting inhibitory or excitatory channel opsins in identified critical excitatory or inhibitory neurons, respectively, proved to stabilize the network activity [195]. However, to be effective, antiepileptic interventions also require an accurate and fast method to detect in advance the unexpected seizure, such that the neuromodulation can be applied in a specific temporal window to prevent the epileptic attack. To this end, closed-loop paradigms have been designed to identify and rapidly modulate, via optogenetic stimulation, the epileptic network [196,197,198]. This paradigm has proved to be able to control the excitability of the epileptic circuits and block the surge of a frank epileptic attack [199,200,201]. Although these experiments targeted specific groups of neurons, recent studies reached an even higher level of cellular specificity identifying a distinct type of cell and the superhubs that appear to control network excitability [202]. This cell type was identified by looking at new patterns of cell–cell connectivity and communication in the data set obtained by whole-brain functional imaging, with cellular resolution, in a validated acute model of epilepsy in zebrafish. Higher order motif-focused network analysis revealed that the instability of the network was associated with a new functional cell type, i.e., the superhub. These cells preferentially emerge in the pre-seizure phase and exhibit an extremely rich feed-forward motif-connectivity with the rest of the network, providing a significant increase in neuronal excitability. Simulated network perturbations revealed that disconnecting superhubs in the pre-seizure phase was significantly more effective in dampening the hyperexcitability and stabilizing network dynamics than disconnecting hub cells, traditionally identified on the basis of functional connectivity. These findings were corroborated by the presence of superhubs in the dentate gyrus of the hippocampus in an established mouse model of epilepsy. Again, disconnecting superhubs in the modeled network of chronic dentate epilepsy was significantly more effective in stabilizing neuronal circuits with respect to modeled disconnection of traditional hubs. Altogether these findings highlight the power of largescale functional recording of neuronal activity along with computational modeling in disentangling epileptic circuits and identifying the critical cellular nodes in control of network excitability. The identification of a single cellular type as “master controller” of the hyperexcitability of epileptic circuits represents a major breakthrough in the understanding of the mechanism underlying epilepsy and opens a completely different perspective in therapeutic interventions, characterized by highly selective and minimally invasive cellular targets for the control of epileptic seizures.

The hyperexcitability which characterizes epileptic networks is known to also be related to the diminished, if not absent, inhibitory GABAergic drive. Electrophysiological recordings from hippocampal and cortical tissue excised from epileptic patients to optimize their treatment revealed that GABA antagonists reduce the frequency of the epileptic seizures, indicating that GABA is excitatory in these chronically epileptic tissues [203,204,205]. This finding has been corroborated by several preclinical studies showing that neurons exhibit a higher Cl intracellular concentration prior to the ictal event [206]. As the polarity (hyperpolarization versus depolarization) of neuronal responses to GABA is critically regulated by the intracellular Cl concentration, measuring the intracellular Cl levels would be critical to dissecting the pathogenesis of the epileptic seizures and designing tailored treatments. Also, in this case, the generation of genetically encoded sensors along with multiphoton imaging have been instrumental to directly measure the intracellular Cl concentration in vivo in large populations of neurons with cellular resolution. These sensors have proved critical to unravelling GABA polarity not only in epilepsy but also in several brain disorders [207,208,209].

### 4.6. Autism Spectrum Disorders 

Autism spectrum disorders (ASD) are complex neurodevelopmental disorders characterized by a wide range of social and cognitive deficits. In addition, hyper-reactivity to sensory stimuli, a common and debilitating symptom, is thought to contribute, if not being causative, to impaired social interactions and cognitive functions [210,211]. The neuronal mechanisms underlying the core features of ASD remain elusive. Large-scale functional imaging with cellular resolution has recently begun to shed light on the neuronal circuits, cell types and network dynamics associated with ASD deficits. Chen et al. [212] exploited a mouse model, i.e., Shank3B^−/−^ to dissect the mechanism underlying sensory alterations in ASD. Shank3B^−/−^ mice exhibit hyper sensitivity to weak stimulation of the vibrissae. When performing calcium imaging with cellular resolution in the somatosensory cortex, Chen et al. found increased spontaneous and sensory-evoked activity in the pyramidal neurons in the somatosensory cortex (SS1), and reduced activity in the GABAergic neurons of the same brain area. Selective deletion of Shank 3B in GABAergic interneurons resulted in hyperexcitability of SS1 pyramidal neurons and hyper sensitivity to vibrissae motion. These data unraveled that GABAergic interneurons altered functionality is responsible for hyperactivity of excitatory neurons and altered sensory perception. These data corroborate a long-standing hypothesis which posits that alterations in development and/or function of inhibitory interneurons are thought to play a critical role and likely represent a common mechanistic link in the heterogenous scenario of ASD [213,214,215].

One of the major limitations in understanding the neuronal basis of hypersensitivity and other core features of ASD is the challenge to investigate the neuronal information processing across the different brain areas involved and dissecting the dynamics of distributed neuronal circuits. A suitable model organism to overcome this limitation is offered by the larvae of zebrafish. The transparency and small size of this organism enable whole-brain functional imaging at cellular resolution, making it possible to investigate the entire pipeline along which neuronal information is elaborated. Combining whole-brain functional imaging along with anatomical location of distinct active neurons [216] identified specific neuronal defects in different brain areas, underpinning hypersensitivity to sounds in fmr 1^−^^/^^−^ larvae. In mutants, auditory-evoked responses were more plentiful and/or stronger in distinct auditory brain areas. In addition, functional connectivity was higher in mutants with respect to the control larvae, whereas the decoding capacity of the distinct component of the ascending auditory pathway was altered.

### 4.7. Migraine

Migraine is a common and debilitating neurological disorder, characterized by a strong head ache preceded by an aura, which most commonly is a sensory hallucination, such as flushing visual percepts, numbness and tingling [217,218]. The mechanisms underlying migraine still remain elusive, although mouse models with the monogenic form of migraine has been valuable in unravelling at least some neurological features associated with migraine. Using iGluSnFR to measure the glutamate release in the barrel cortex in response to sensory stimulation, it was found that mice carrying a familial hemiplegic migraine type 2 mutation (FHM2) exhibited a slower glutamate clearance [219]. Surprisingly, FHM2 mice exhibit also spontaneous release of glutamate, which was not observed in controls [220]. In addition, this study was able to establish a causal relationship between the slower glutamate clearance, the aberrant glutamate releases (indicated as glutamate “plumes”) and the onset of spreading depression (SD). By recording the neuronal population activity by means of two-photon imaging, it was possible, for the first time, to follow SD with cellular resolution. This approach enabled identification of the brain area with higher glutamate fluorescence and high plumes frequency as the point of initiation of SD. Plumes indeed precede SD and do not coincide with SD itself. Blocking glutamate plumes was shown to prevent SD initiation. This higher concentration of glutamate was due to a slower clearance of glutamate in FHM2 mice with respect to controls. Indeed, by blocking the glutamate reuptake, WT mice exhibited higher plume frequency. Altogether, this study unraveled a new mechanism for migraine related to an unbalance between release and uptake of glutamate that results in higher concentrations of glutamate in the extracellular space. This condition is responsible of a massive depolarization of neurons (SD) that could occur not only in migraine but also in several other neurological disorders [220]. The research is now focused on understanding which other disorders could exploit such a mechanism.

### 4.8. Depression

Animal models mimicking depression-like phenotypes most frequently involves challenging mice with stressors in chronic or acute paradigms, relying on the well-established relationship between stress and depression in humans [221,222]. Social defeat and chronic mild stress behavioral tests [223] were successfully paired to optogenetic approaches targeting different brain regions in order to test their involvement in the expression, suppression or modulation of depression-like symptoms. Ventral tegmental area dopaminergic neurons (VTA-DA) are critically involved in processing reward prediction error cues, and are thus deeply involved in the underlying mechanisms, such as depression-like phenotypes [224]. VTA-DA neurons display a modulation of typical firing patterns in mice subjected to chronic social defeat stress and expressing depression-like behaviors in response, contrary to control mice or animals resilient to the stress challenge. VTA-DA cells show a decreased phasic firing in comparison [225,226]. Indeed, optogenetically increasing VTA-DA firing rates leads to increased social avoidance and reduced reward seeking in otherwise stress-resilient mice [227]. Additionally, optogenetic selective activation of VTA-DA neurons projecting to nucleus accumbens recapitulates bulk VTA-DA stimulation while specifically targeting VTA-DA cells projecting to medial prefrontal cortex (mPFC), promoting resiliency. On the other hand, optogenetic inhibition of VTA-mPFC pathways triggers social withdrawal, which is recognized as a marker for depression [228]. Different stressors, however, may have different impacts on the VTA-DA activity; hence, optogenetic manipulations might trigger context-specific effects [229]. Another key structure involved in the expression of depression-like phenotypes induced by stressor exposure is obviously the hippocampal formation (HF) and connected regions such as the basolateral amygdala (BLA) [230]. Activity-dependent targeting of stress-associated engrams in the hippocampus with optogenetic tools allowed for the reactivation of local neuronal circuits associated with the stressor memory leading to increased resiliency [231,232]. The effect of the optogenetic stimulation was blocked upon BLA inhibition and was demonstrated to depend upon HF-NAc communication. Both in humans and in mouse models, mPFC hypoactivity is described in the context of depression-like behaviors [233,234]. Indeed, optogenetic stimulation of mPFC in stress-challenged mice reduces depression-like behaviors [233,235], although different aspects of behaviors are affected, targeting different parts of mPFC [236,237]. In general, recent optogenetic approaches in this field suffer from the relatively restricted repertoire of experimental paradigms, suitable animal models and functional data concerning network activity in depression-like scenarios [235,238]. From the functional perspective, in a recent study [239], iGluSnFR were used in a mesoscale imaging longitudinal approach to dissect spontaneous glutamatergic activity in mice subjected to chronic social defeat stress. In this case, a marked decrease in functional connectivity was observed and confirmed by voltage imaging in stressed animals compared with controls. Interestingly, both depression-like behaviors and functional hypoconnectivity were transiently alleviated with a subanesthetic ketamine administration.

### 4.9. Application of Optical Methods to Non-Human Primates

Non-human primates (NHPs) are in many respects more similar to human beings than other animal models, including behavioral, social and cognitive aspects related to so- called higher cognitive functions. Exploring disease mechanisms impacting on such “human-like” phenotypes, such as neuropsychiatric disorders or neurodevelopmental and cognitive dysfunctions using these animal models is extremely valuable [240,241,242,243,244,245]. Nevertheless, adapting optical tools to probe NHPs neurophysiology in conscious and receptive animals proved very challenging [246,247,248,249]. First of all, until recently, viral transduction resulted in much less efficiency compared to other animal models, and additionally large movement artefacts resulted in highly detrimental imaging quality in head restrained NHPs, and optical access to brain structures proved to be less stable in time due to strong tissue reaction to the implantation of optical windows. In [250] and in [251], several technical improvements enabling long term optogenetic and imaging (respectively) approaches in NHPs, providing solid background for further experimental investigations, corroborated by electrophysiological and histological data are described. In [252], a method combining two-photon optogenetics with one photon wide field or two-photon imaging in NHP visual cortex providing evidence for stable recordings of visually or optogenetically evoked visual activity and related behaviors is described. Recently, a transgenic Callirtix NHP line was established using a lentiviral approach to deliver GCaMP5g or GCaMP6s under the human synapsin promoter to embryos and achieving both reporter expression in mature neurons and efficient germline transmission of the transgene [253]. Alternatively, recent developments allowed for the transduction of GCaMP6f in NHPs using adapted rAAV for long-term, one-photon functional imaging in conscious and receptive animals [247,254].

## 5. Conclusions

The recent and constant innovations in the field of imaging cellular activity in vivo and in the use of light to control cellular dynamics provide powerful and unparallel tools to collect functional data from large populations of neurons and unravel the contribution of the different components of the brain circuits in the functionality or dysfunctionality of a brain mechanism. Along with being a valuable resource in basic research investigation, the developed approaches hold an enormous potential for a more detailed characterization of the alterations associated with neurodegenerative, neurological and psychiatric disorders essential to design-effective treatments. 

## Figures and Tables

**Table 2:** Principal depolarizing and hyperpolarizing opsins. For each element, ion specificity, spectral peak and decay time (tau-off) are reported. The values have been obtained from the references.

	Opsins	Ions		Spectral Peak (nm)	Tau Off (ms)	References
		Influx	Efflux			
**Depolarizing**	ChR2	Na^+^	-	470	10	[126]
	CoChR	Na^+^	-	470	30	[127]
	Chronos	Na^+^	-	530	3.6	[127]
	ChroME	Na^+^	-	530	3	[128]
	ChroMEs	Na^+^	-	530	13	[129]
	ChroMEf	Na^+^	-	530	9.6	[129]
	ChRmine	Na^+^	-	585	2	[130]
	ChrimsonR	Na^+^	-	590	15.8	[127]
	f-Crimson	Na^+^	-	590	5.7	[131]
	vf-Crimson	Na^+^	-	590	2.7	[131]
**Hyperpolarizing**	GtACR2	Cl^-^	-	480	40	[132]
	GtACR1	Cl^-^	-	520	15	[128,132]
	Arch	-	H^+^	570	20	[133]
	eNpHr3.0	Cl^-^	-	590	40.5	[134]
	AIACR1	Cl^-^	-	590	90	[135]

## Data Availability

Not applicable.
